# Burnout in the intensive care unit professionals: A systematic review

**DOI:** 10.1097/MD.0000000000005629

**Published:** 2016-12-16

**Authors:** Chien-Huai Chuang, Pei-Chi Tseng, Chun-Yu Lin, Kuan-Han Lin, Yen-Yuan Chen

**Affiliations:** a Department of Medical Education, National Taiwan University Hospital; b National Taiwan University College of Medicine; c Graduate Institute of Medical Education & Bioethics, National Taiwan University College of Medicine, Taipei, Taiwan.

**Keywords:** burnout, depersonalization, emotional exhaustion, intensive care, personal accomplishment

## Abstract

**Background:**

Burnout has been described as a prolonged response to chronic emotional and interpersonal stress on the job that is often the result of a period of expending excessive effort at work while having too little recovery time. Healthcare workers who work in a stressful medical environment, especially in an intensive care unit (ICU), may be particularly susceptible to burnout. In healthcare workers, burnout may affect their well-being and the quality of professional care they provide and can, therefore, be detrimental to patient safety. The objectives of this study were: to determine the prevalence of burnout in the ICU setting; and to identify factors associated with burnout in ICU professionals.

**Methods:**

The original articles for observational studies were retrieved from PubMed, MEDLINE, and Web of Science in June 2016 using the following MeSH terms: “burnout” and “intensive care unit”. Articles that were published in English between January 1996 and June 2016 were eligible for inclusion. Two reviewers evaluated the abstracts identified using our search criteria prior to full text review. To be included in the final analysis, studies were required to have employed an observational study design and examined the associations between any risk factors and burnout in the ICU setting.

**Results:**

Overall, 203 full text articles were identified in the electronic databases after the exclusion of duplicate articles. After the initial review, 25 studies fulfilled the inclusion criteria. The prevalence of burnout in ICU professionals in the included studies ranged from 6% to 47%. The following factors were reported to be associated with burnout: age, sex, marital status, personality traits, work experience in an ICU, work environment, workload and shift work, ethical issues, and end-of-life decision-making.

**Conclusions:**

The impact of the identified factors on burnout remains poorly understood. Nevertheless, this review presents important information, suggesting that ICU professionals may suffer from a high level of burnout, potentially threatening patient care. Future work should address the effective management of the factors negatively affecting ICU professionals.

## 1 Introduction

Burnout has been described as a prolonged response to chronic emotional and interpersonal stress on the job^[1]^ that is often the result of a period of expending excessive effort at work while having too little recovery time.^[2]^ Burnout was first described in 1974 by Freudenberger, thereby inspiring the investigation of the characteristics and prevalence of this phenomenon.^[3]^ Maslach and Jackson defined burnout as having three different aspects: emotional exhaustion, depersonalization, and lack of personal and professional completion.^[4]^

Burnout has been recognized as an occupational hazard in various people-oriented professions, including healthcare.^[5]^ To construct a resilient health system, it is important that burnout in healthcare workers be addressed.^[6]^ Burnout in healthcare workers may affect healthcare worker well-being and the quality of professional care they provide^[7]^ and can, therefore, be detrimental to patient safety.^[8]^ The prevalence of burnout in healthcare workers is among the highest out of the occupations that have been surveyed^[9]^ (occupational physicians 11%, psychiatrist 9%, general practitioners 8%, community nurses 8%, and midwives 7%). Workload and time pressure have been cited as the major causes of high levels of burnout, with both qualitative and quantitative workload contributing to burnout, especially in the emotional exhaustion dimension.^[10]^ Patient-related stressors, experience, personality, and work-related attitudes have also been identified factors associated with burnout.^[11]^ Burnout may also affect both the physical and psychological health of the healthcare worker.

The composition of intensive care units is unique in the spectrum of healthcare services provided. Medical care is provided in intensive care units by a critical care team, composed of intensivists, critical care nurses, respiratory therapists, pharmacists, dietitians, and other medical professionals. Patients with any life-threatening illnesses may be admitted to the intensive care unit. The mortality rate in critical care patients have been reported to range from 10% to 29%.^[12]^ For medical professionals working in the intensive care unit (ICU), discrepancies in job demands, responsibility overload, end-of-life issues, and interpersonal conflict all constitute potential stressors.^[13]^ Healthcare workers are particularly susceptible to burnout,^[9]^ which has been observed to occur at an especially high rate in this population, with at least 20% of ICU professionals scoring high on burnout indicators.^[13]^

As a recent World Health Organization (WHO) reports emphasized, research, and evaluation are important in developing health policies and creating comprehensive health systems,^[6,14,15]^ and the health of medical personnel should be not excluded from this principle, especially in the era of universal health coverage.^[15]^ While a recent systematic review reported the prevalence of burnout in the ICU and several effective strategies to prevent burnout among ICU professionals,^[16]^ the true magnitude of burnout remains open for discussion. Additionally, an important literature gap still exists in terms of the risk factors for the development of burnout in ICU professionals. Therefore, the purposes of our review are to determine the prevalence of burnout and to identify risk factors associated with burnout in ICU professionals.

## 2 Methods

### 2.1 Literature search

The PubMed, MEDLINE, and Web of Science electronic databases were systematically searched in June 2016. The terminology used in this review was utilized to identify Medical Subject Headings (MeSH) and free-text terms, and “burnout” and “intensive care unit” were used as search terms to identify potentially relevant studies. The original articles for observational studies (cross-sectional, cohort, and case-control studies) were eligible for inclusion if they were published in the English language between 1996 and June 2016. Qualitative studies, reviews, clinical treatment trials, case reports and series, cadaveric studies, biomechanical studies, and laboratory studies were excluded. The references of all relevant articles were also screened for additional publications.

### 2.2 Study selection

Each publication was initially assessed for relevance using data presented in the abstract. When the abstract failed to provide sufficient information, a reprint of the full text was obtained. Two reviewers (CHC, PCT) independently evaluated the abstracts identified using our search criteria and selected eligible articles for full text review. Full text articles were screened for eligibility according to predefined criteria. To be included in the final analysis, studies were required to have employed an observational study design to examine the associations between any risk factors and burnout in the ICU setting. Studies were excluded if they recruited non-ICU professionals and burnout was not included as an outcome variable.

### 2.3 Quality assessment

Two reviewers independently assessed the quality of each study using a modification of the checklist designed by Downs and Black^[17]^ and Crombie.^[18]^ Checklists were modified according to the type of study reviewed.^[19]^ Two reviewers performed independent, in-depth reviews of each eligible study. The results of these reviews were compared using the kappa statistic to measure the level of agreement between the two reviewers. Values of kappa between 0.40 and 0.59 were considered to reflect fair agreement, while values between 0.60 and 0.74 were considered to reflect “good” agreement, and values of 0.75 or more were considered to reflect “excellent” agreement.^[20]^ The interrater reliability of the two reviewers’ checklist scores was evaluated using type 2, 1 intraclass correlation coefficients (ICCs). When the scores for any article differed between the two reviewers, a consensus score was assigned after comprehensive discussion.

Each checklist item were scored as “yes,” “no” or “unable to determine” where unclear or insufficient information was provided on a specific criterion. Positively scored criteria were added in order to obtain a total quality score for each paper. The maximum obtainable scores for each paper were 20 points for cohort studies and 19 points for other studies. The results were expressed as percentages of the total attainable score.

### 2.4 Data extraction and synthesis

For each paper, the following information was extracted: year of publication, country of study, setting, assessment tool for burnout measurement, sample size, participation rate, burnout prevalence, and risk factors. Data were extracted independently by 2 reviewers. This systematic review used a narrative synthesis format to determine the prevalence of burnout and to identify risk factors associated with burnout in ICU professionals.

Ethical approval was not requested for this study as it did not involve human participants.

## 3 Results

**Figure 1:**
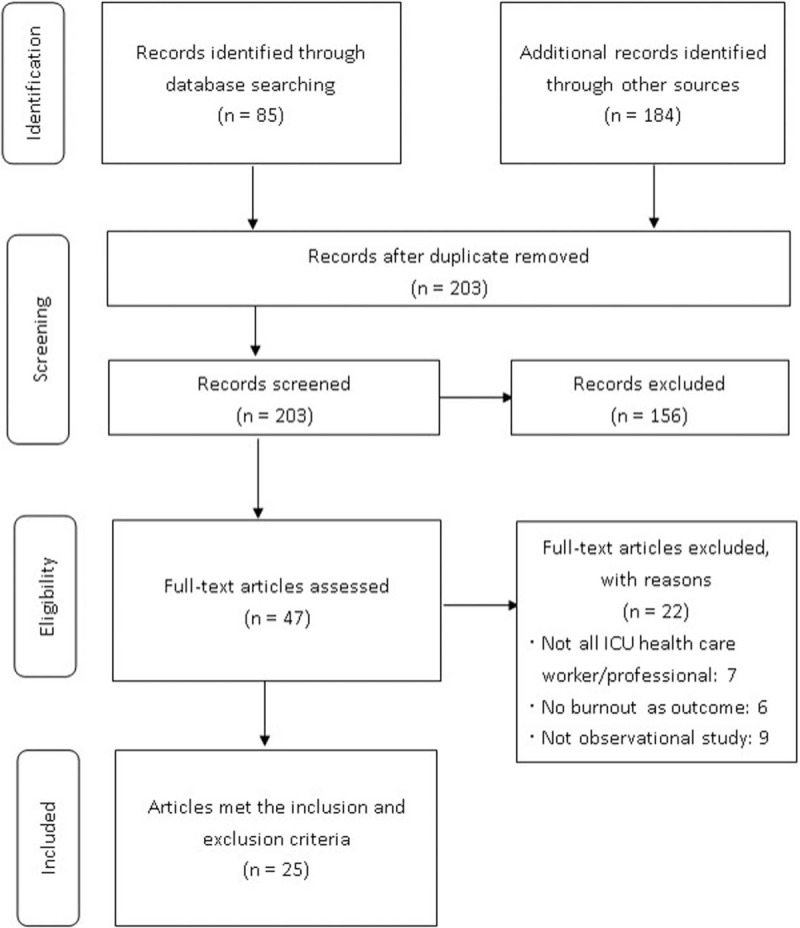
Flow diagram of the literature search process.

A total of 203 full texts were identified from the PubMed, MEDLINE, and Web of Science electronic databases, and after screening for duplicate articles and performing the initial review, 25 studies fulfilled our inclusion criteria. We excluded texts in which not all participants were ICU healthcare workers or professionals, where the outcome was “no burnout”, and that were not an observational study. The review process is described in Fig. 1.

The quality of the studies by checklist item is presented in Table 1. There was a high level of agreement in the assessments of the included articles performed by the two reviewers (Kappa 0.82), and the intraclass correlation coefficient for the interrater reliability of the total checklist scores for individual raters was 0.996 (95% confidence interval [CI] 0.989–0.998). The characteristics of the selected articles are presented in Table 2   , including the year of study, country, setting, burnout measurement, sample size (participation rate), prevalence of burnout, and risk factors (risk or protective factors).

**Table 1:**
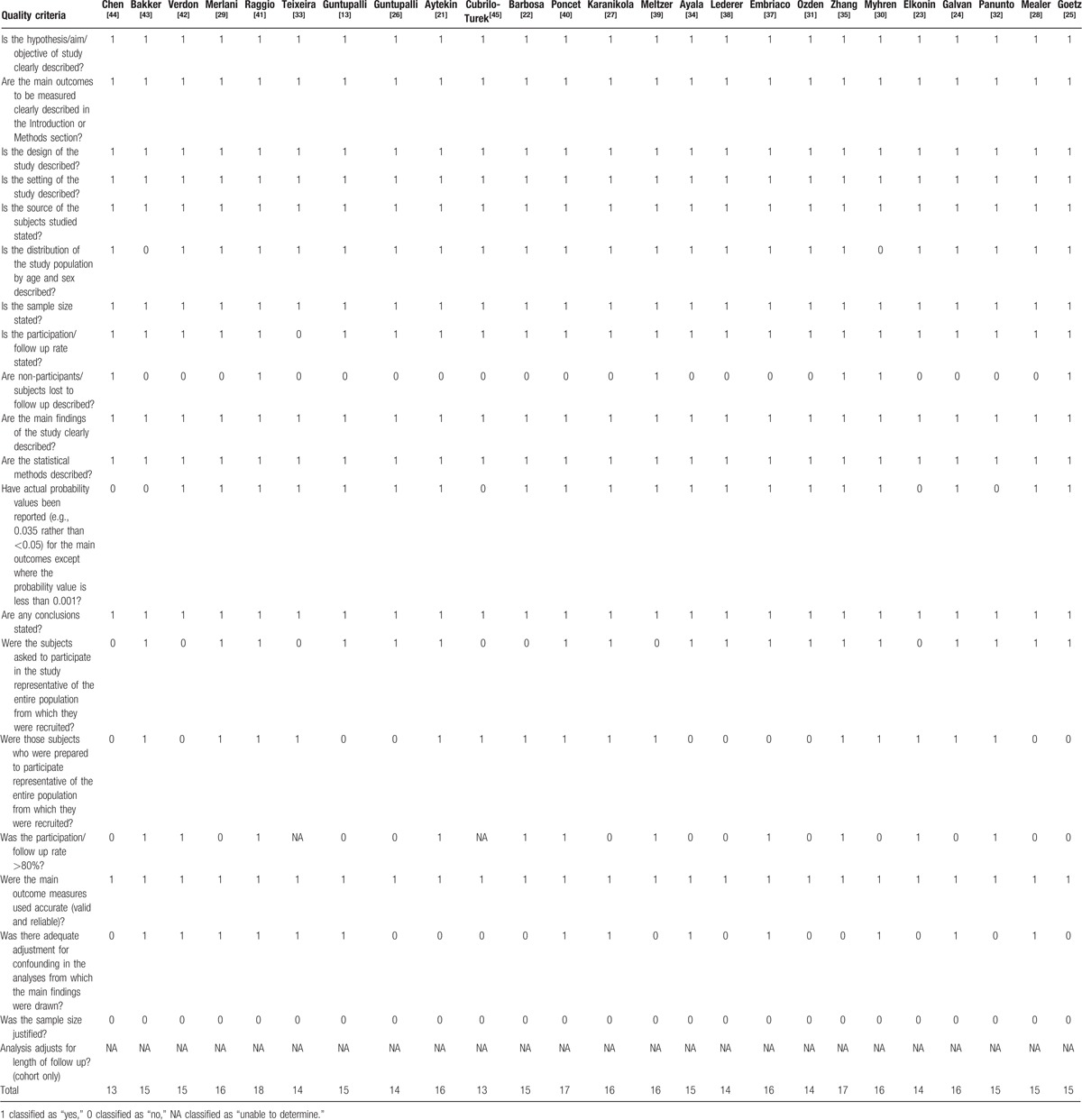
Quality of papers (n = 25).

**Table 2:**
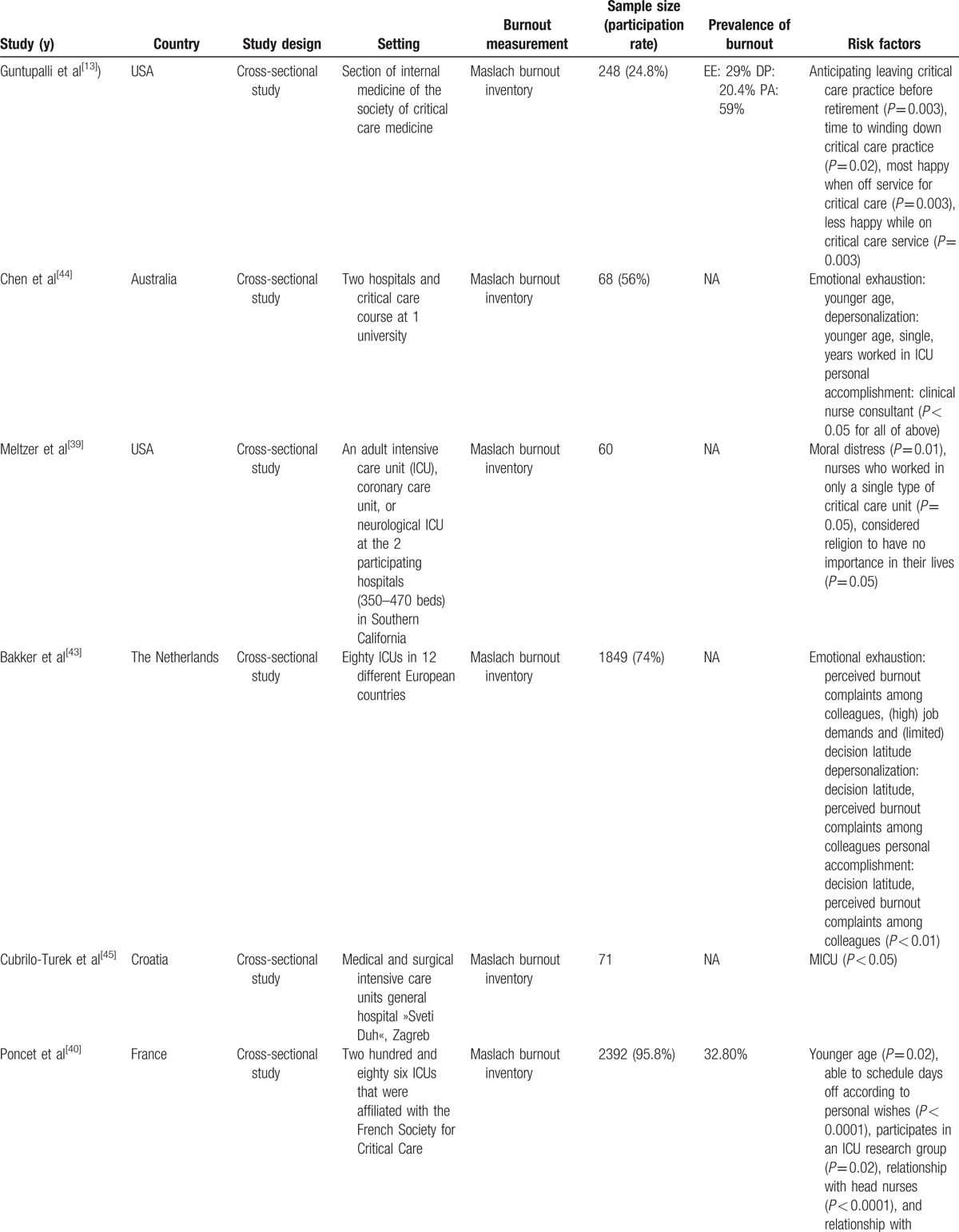
Characteristic of included studies.

**Table 2 (Continued):**
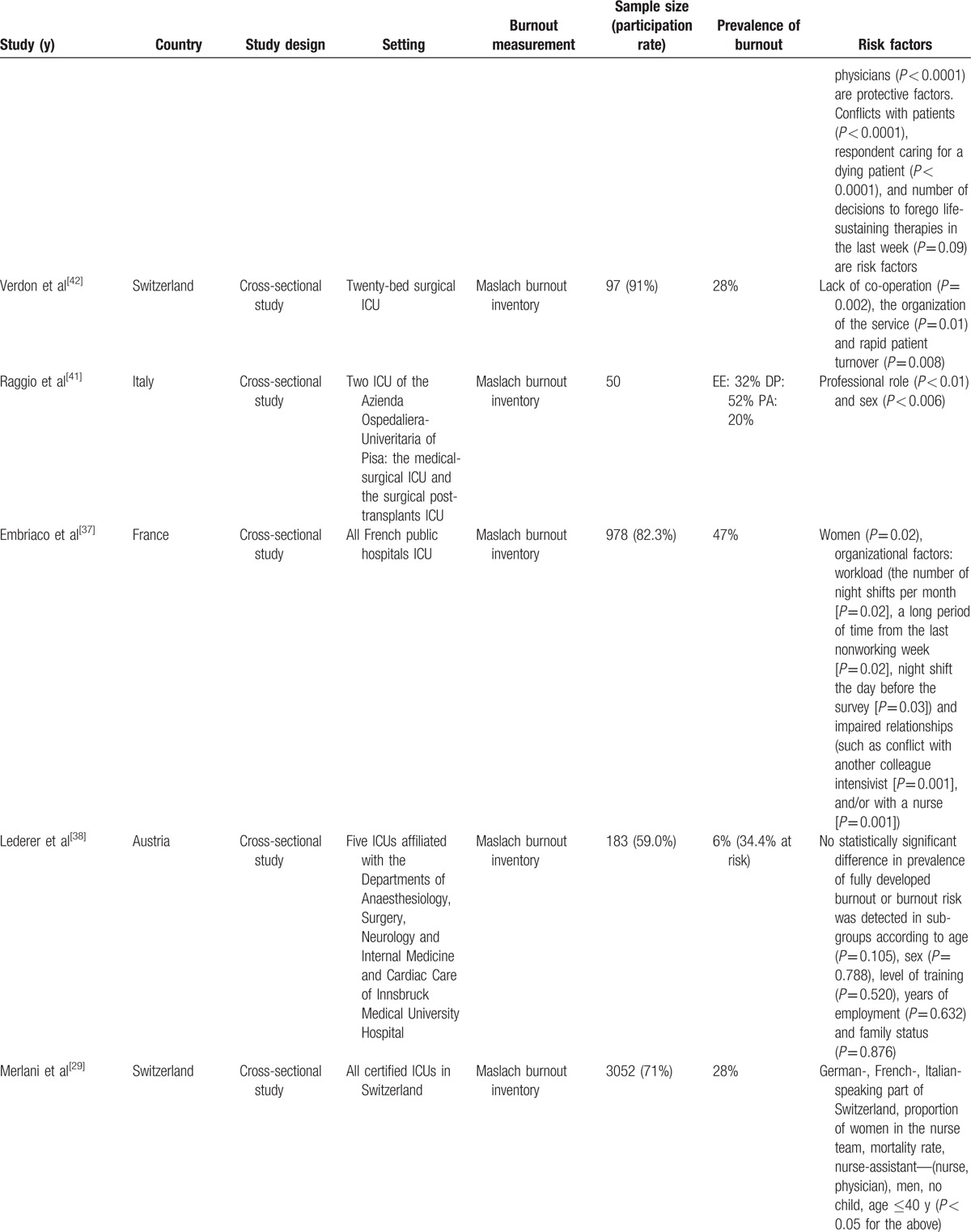
Characteristic of included studies.

**Table 2 (Continued):**
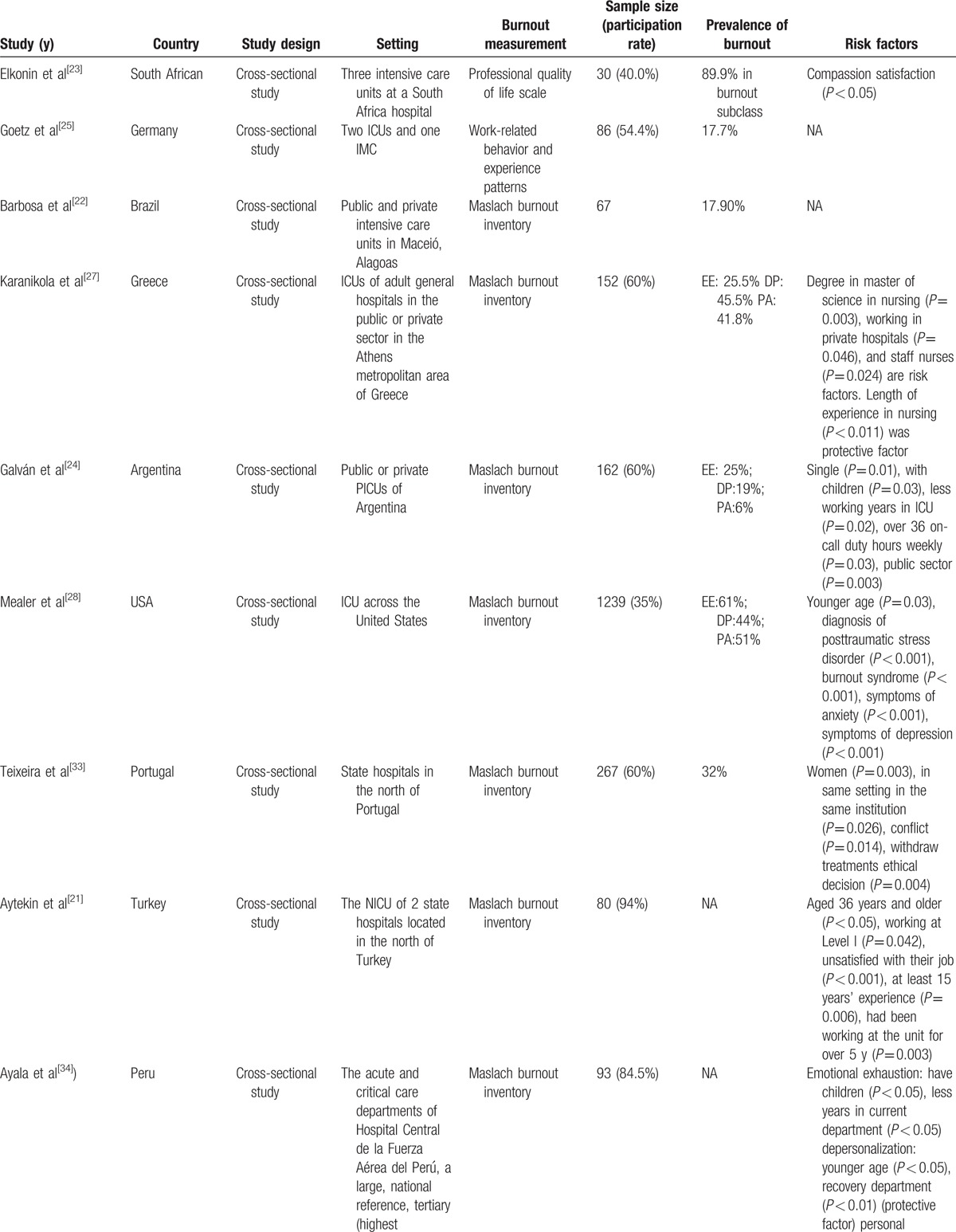
Characteristic of included studies.

**Table 2 (Continued):**
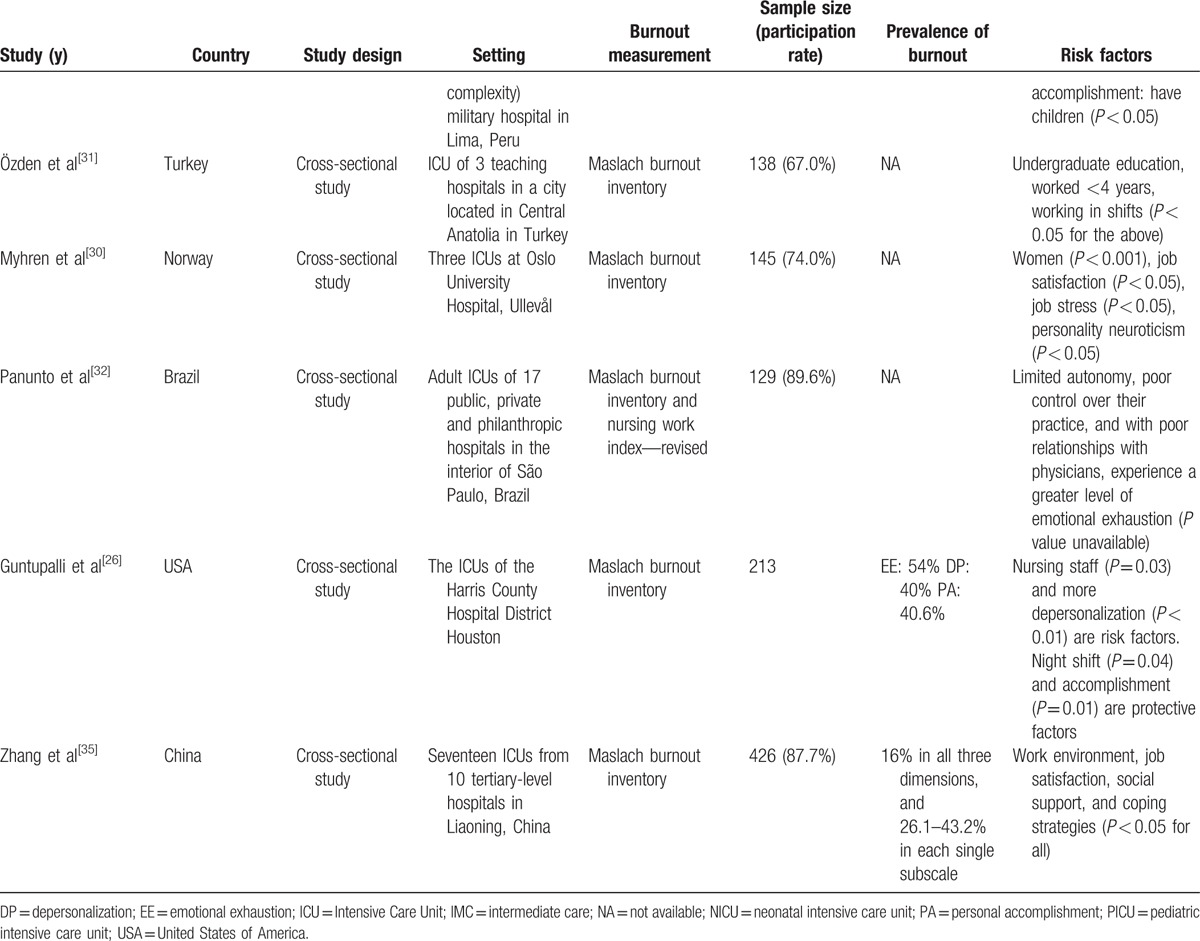
Characteristic of included studies.

Regarding the year of publication, 60% of the articles were published after 2011.^[21–35]^ Only one article was published before 2000.^[36]^ Regarding the journal field, of the included studies, 12 were published in the critical medicine field,^[26,27,29,30,33,36–42]^ 7 in were published the nursing field,^[21,25,27,28,31,43,44]^ 2 were published in the field of ethics,^[31,34]^ and 1 was published in the anthropology field.^[45]^ The geographical distribution analysis showed that of the included studies, 12 were conducted in the European Union (Netherland, Croatia, France, Switzerland, Italy, Austria, Germany, Greece, Portugal, Norway),^[25,27,29,30,33,37,38,40–43,45]^ 4 were conducted in the USA,^[26,28,36,39]^ 4 were conducted in South America (Brazil, Argentina, Peru),^[22,24,32,34]^ 2 were conducted in Turkey,^[21,27]^ 1 was conducted in South Africa,^[23]^ 1 was conducted in Australia,^[44]^ and 1 was conducted in China.^[35]^

A variety of instruments were utilized for data collection. The most commonly used instrument in quantitative studies was the Maslach burnout inventory (MBI). One study combined the MBI with the nursing work index-revised.^[32]^ Additionally, the professional quality of life scale^[23]^ and work-related behavior and experience patterns^[25]^ were used in the selected articles.

Among the studies, there were 10 articles that assessed all personnel working in the ICU,^[22,26,29,30,33,37,38,40,41,45]^ while 13 studies assessed nurses only,^[21,23,25,27–29,32,34,35,39,42–44]^ and 2 studies assessed physicians only.^[24,36]^ Except for 1 study that did not report sex data,^[21]^ the selected studies had a preponderance of women personnel, with only 2 studies having a man majority.^[37,41]^ One of these studies was targeted exclusively at internal medicine intensivists,^[36]^ and the other was a French nationwide study^[40]^ that comprehensively assessed interns, residents, fellows, and attending physicians.

Among the 5 largest research studies included in our review, the sample size ranged from 978 to 3052.^[29,37]^ Among the other included studies, the largest sample size was 426,^[35]^ and the smallest sample size was 30.^[23]^ Overall, the response rate ranged from 24.8% to 96%.^[36,40]^

### 3.1 Prevalence of burnout among ICU personnel

The prevalence of burnout among ICU personnel in the selected studies varied from 6% to 47%. Some studies presented the prevalence of the 3 domains of MBI separately.^[24,26–28,35,36,41]^ In the emotional exhaustion domain, the prevalence of burnout ranged from 25.0% to 61.0%, while the prevalence of burnout in the depersonalization domain 19.0% to 45.5%, and the prevalence of burnout in the personal accomplishment domain ranged from 6.0% to 59.0%.

Of note, 4 of the 5 largest research studies reported prevalence data.^[28,29,37,40]^ The prevalence rates of burnout were 32.8% and 47% in 2 different nationwide French studies^[37,40]^ and 28% in Switzerland.^[29]^ In the study conducted in the United States, the prevalence rate of burnout was 61% in the emotional exhaustion (EE), 44% in the depersonalization (DP), and 51% in the personal accomplishment (PA) domain.^[28]^

### 3.2 Risk factors for burnout among ICU personnel

The burnout risk factors identified in the review articles were mainly related to age, sex, working experience in an ICU, working experience in nursing, working environment, organizational factors, interpersonal relationships with colleagues, personality traits and beliefs, workload and shift work, marital status, and educational degree.

#### 3.2.1 Age

In our review, 6 studies indicated that young age was a risk factor for burnout.^[21,28,29,33,40,44]^ Chen and McMurray and Ayala and Carnero reported, more specifically, that young age was a risk factor for burnout in the domains of depersonalization and emotional exhaustion.^[34,44]^ More specifically, Merlani et al^[29]^ reported that being younger than 40 years old was a risk factor for burnout. Furthermore, Aytekin et al^[21]^ identified a lower prevalence of burnout in the personal accomplishment domain among those aged 36 years and older (*P* < 0.05). Additionally, Mealer et al^[28]^ noted that an increase in age was significantly associated with high resilience among ICU nurses (*P* = 0.03) in a study that was conducted using the Connor–Davidson Resilience Scale.

#### 3.2.2 Sex

Sex was reported as a risk factor for burnout among ICU personnel in 4 cross-sectional studies.^[29,33,37,41]^ Raggio and Malacarne^[41]^ reported that in men, particularly men doctors, a high degree of DP was observed, while in women physicians, the tendency towards EE was much higher. In the 1-day nationwide survey conducted in France by Embriaco et al,^[37]^ the univariate analysis showed a higher prevalence of burnout in women intensivists. However, a large Swiss multicenter study showed having a higher proportion of women nurses on the working team was associated with a decreased risk of burnout.^[29]^ The author also indicated that men sex was a caregiver-related factor associated with high risk of burnout.

#### 3.2.3 Marital status and child bearing

Four studies included in our review showed consistent results regarding familial status, suggesting that being single and childless might be associated with a higher risk of burnout.^[24,29,34,44]^ Chen and McMurray^[44]^ indicated that among nurses, being married was associated with lower levels of burnout in the depersonalization domain. Similarly, an investigation of ICU professionals in Portugal indicated that a higher level of burnout was associated being single and without children.^[33]^

#### 3.2.4 Work experience in nursing and the ICU

Less working experience was found to be associated with different aspects of burnout in several cross-sectional studies. In an Argentinian study, Galván et al^[24]^ reported that having worked fewer years in an ICU was a statistically significant risk factor for burnout among ICU personnel. Additionally, Aytekin et al^[21]^ found that having worked fewer years in the nursing field was a significant risk factor for burnout in the PA domain. A Greek study showed that experience (years) in nursing was inversely correlated with burnout in the DP domain (*r* = 0.214, *P* < 0.011), but not with burnout in the EE and PA domains (*P* > 0.6).^[27]^ Özden et al^[31]^ reported that nurses that had worked for less than 4 years had higher mean scores in the EE and DP domains; however, this result was not statistically significant.

#### 3.2.5 Organizational factors

Regarding organization factors, in the study conducted by Poncet et al,^[40]^ 2 organizational factors were associated with burnout level: 1 was participation in an ICU research group (OR 0.74; CI, 0.56–0.97; *P* < 0.03) and the other was the ability to choose days off according to personal wishes (odds ratio [OR], 0.69; CI, 0.52–0.91; *P* < 0.009).

#### 3.2.6 Night shifts and working hours

Two studies noted that working night shifts and number of working hours were factors associated with burnout. Galván et al^[24]^ found that being on-call for more than 36 hours a week increased the risk of burnout risk. A nationwide French study indicated that an increased frequency of night shifts per month and the time duration since the last non-working week were both associated with increased burnout risk, as was having a night shift the day before conducting the survey.^[40]^

#### 3.2.7 Ethical issues and end-of-life decision-making

Embriaco et al^[37]^ and Poncet et al^[40]^ reported an increased prevalence of burnout among physicians and nurses who had often been dealing with death or who had participated in decisions of foregoing life-sustaining therapy. Teixeira et al^[33]^ also found that ethical decision-making regarding end of life issues (including the decision to withhold or withdraw treatment) was positively associated with the observed level of burnout.

#### 3.2.8 Personality and traits

In a study conducted in Norway, Myhren et al^[30]^ used the basic character inventory methodology, which is composed of 3 dimensions of, neuroticism, extroversion, and control/compulsiveness, to evaluate burnout. The results showed that higher burnout scores were associated with having a “vulnerable” personality. Similar findings were reported by Mealer et al,^[28]^ who indicated that psychological resilience was independently associated with a lower prevalence of posttraumatic stress disorder and burnout syndrome among intensive care unit nurses.

## 4 Discussion

### 4.1 Prevalence: comparison of burnout prevalence between ICU and non-ICU healthcare workers

Environment influences health,^[46]^ and the intensive care unit, being a totally different environment from general wards, has been found to be associated with higher prevalence of burnout due to its associated increased work intensity, much higher degree of difficulty with regards to patient disease status, and imposition of higher emotional stress on both family members and patients. Although the prevalence of burnout in the ICU healthcare workers assessed in included studies varied widely (from 6% to 47%), the 4 large-scale research studies reported burnout prevalence rates ranging from 28%∼61%, suggesting that ICU healthcare workers were slightly more prone to burnout than average health care workers.

A study comparing pediatricians, cancer physicians, and general practitioners showed that approximately 1/3 of the physicians had burnout, and general practitioners had the highest burnout prevalence, with 36% of general practitioners having high EE scores, 36% of general practitioners having high in DP scores, 15% of general practitioners having low in PA scores.^[47]^ Similarly, a study conducted in Madrid revealed that 69.2% of primary care physicians had moderate to high levels of burnout,^[48]^ which was much higher than that of the average physician. Thus, it appears that, along with emergency nurses and oncologists, ICU health workers exhibit a high prevalence of burnout.

### 4.2 Risk factors for ICU burnout

#### 4.2.1 Individual variables/sociodemographic characteristics

##### 4.2.1.1 Age

Pooled analyses performed in several previous studies have shown that caregiving professional age was inversely associated with burnout, which is consistent with the results found in the present study.^[34,49]^ It is believed that younger individuals may be more sensitive to job burnout.^[50]^ It is possible that caregivers with less seniority are still learning to cope with high workload demands when faced with stressors and less able to schedule days off or asked to work more night shifts, which may have led to the burnout observed in younger caregivers.^[29,44,51]^

##### 4.2.1.2 Length of work experience

Previous studies have revealed different finding regarding the association between burnout level and years of working experience. Some results have indicated that experienced nurses become more skilled and committed to their work, therefore staying more calm and controlled when facing unpredictable situations and feeling more successful in their profession, resulting in the identification of a decreased level of burnout in more experienced professionals.^[30,52–55]^

##### 4.2.1.3 Personality trait

Studies have revealed that personality characteristics may be predictive of burnout^[56–58]^ because personality traits may be associated with problem solving and coping strategies and relate to how well an individual reacts to stressful situations in his or her workplace. Some studies have indicated that job holders with neuroticism were more likely to push themselves hard in their work, resulting in an increased level of burnout.^[59,60]^ Neuroticism has been viewed as a “negative affectivity,”^[61]^ as it may be correlated with increased psychological distress associated with worrying about poor career achievement and, thus, may cause mental health problems.^[57,60,62]^ In line with the aforementioned literature, this review found that neuroticism was strongly associated with burnout.

#### 4.2.2 Occupational factors

Excessive workload and overtime are commonly reported by professionals who work in healthcare systems,^[63]^ especially nurses.^[64–66]^ The backbreaking workload shouldered by nurses is associated with the unpredictable nature of their jobs, and nurses often work understaffed and rotating shifts.^[67]^ Work overload contributes to burnout by depleting the capacity of the people available to meet the demands of the job,^[5]^ which was described by several of the included studies^[24,31,37]^

### 4.3 Consequences of burnout

A higher level of burnout among healthcare professionals has been reported to be associated with negative outcomes, such as psychological and other types of discomfort,^[42]^ higher staff turnover, lower job satisfaction, and heart disease.^[7]^ Additionally, having a sedentary occupation, such as working in an ICU, has been shown to be associated with the development of metabolic syndrome, putting workers at increased risk for diabetes, cardiovascular events, and coronary heart disease mortalities.^[68–70]^ As a result, not only may burnout decrease the physical and psychological conditions of healthcare professionals, but it also may harm the health care institutions at which they are employed.

### 4.4 Limitations

Although our review used a comprehensive search strategy, limitations should be noted. First, even though the most important objective of a systematic review is to locate all original reports on the topic of interest, only articles published in the English language were included in this study. This may have introduced bias, but a lack of resources precluded the translation of texts from other languages into the English language. Second, another limitation may be publication bias. Studies that have statistically significant results are more likely to be published than are studies that do not have significant findings. Nevertheless, due to a lack of resources, only references obtained based on an electronic search and review of references included in the available articles were used in this review. Third, one of the requirements of a systematic review is for independent data extraction to be performed by 2 reviewers. In this systematic review, a comparison of the data extracted by 2 assessors was performed and found to be satisfactory.

## 5 Conclusion

In summary, the prevalence rates of burnout among ICU professionals ranged from 6% to 47%. Several risk factors, such as age, sex, marital status, personality traits, work experience in an ICU, work environment, workload and shift work, ethical issues, and end-of-life decision making were found to affect the prevalence of burnout among ICU professionals. However, impact of these risk factors on burnout remains poorly understood. Nevertheless, this review discussed important findings suggesting that ICU professionals suffer from a high level of burnout, which may, in turn, threaten patient care. We believe that burnout in the ICU settings should be considered an important issue in clinical research. Future work should address effective management of the identified risk factors that negatively affect ICU professionals.
